# Gene burden analysis identifies genes associated with increased risk and severity of adult-onset hearing loss in a diverse hospital-based cohort

**DOI:** 10.1371/journal.pgen.1010584

**Published:** 2023-01-19

**Authors:** Daniel Hui, Shadi Mehrabi, Alexandra E. Quimby, Tingfang Chen, Sixing Chen, Joseph Park, Binglan Li, Regeneron Genetics Center, Penn Medicine Biobank, Michael J. Ruckenstein, Daniel J. Rader, Marylyn D. Ritchie, Jason A. Brant, Douglas J. Epstein, Iain Mathieson

**Affiliations:** 1 Department of Genetics, Perelman School of Medicine, University of Pennsylvania, Philadelphia, Pennsylvania, United States of America; 2 Department of Otolaryngology, University of Michigan, Ann Arbor, Michigan, United States of America; 3 Department of Otolaryngology–Head and Neck Surgery, University of Pennsylvania, Philadelphia, Pennsylvania, United States of America; 4 Department of Biomedical Data Science, Stanford University, Stanford, California, United States of America; 5 Regeneron Genetics Center, Tarrytown, New York, United States of America; 6 Institute for Translational Medicine and Therapeutics, Perelman School of Medicine, University of Pennsylvania, Philadelphia, Pennsylvania, United States of America; 7 Department of Medicine, Perelman School of Medicine, University of Pennsylvania, Philadelphia, Pennsylvania, United States of America; 8 Institute for Biomedical Informatics, Perelman School of Medicine, University of Pennsylvania, Philadelphia, Pennsylvania, United States of America; 9 Department of Otolaryngology–Head and Neck Surgery, Corporal Michael J. Crescenz VAMC, Philadelphia, Pennsylvania, United States of America; University of California San Diego, UNITED STATES

## Abstract

Loss or absence of hearing is common at both extremes of human lifespan, in the forms of congenital deafness and age-related hearing loss. While these are often studied separately, there is increasing evidence that their genetic basis is at least partially overlapping. In particular, both common and rare variants in genes associated with monogenic forms of hearing loss also contribute to the more polygenic basis of age-related hearing loss. Here, we directly test this model in the Penn Medicine BioBank–a healthcare system cohort of around 40,000 individuals with linked genetic and electronic health record data. We show that increased burden of predicted deleterious variants in Mendelian hearing loss genes is associated with increased risk and severity of adult-onset hearing loss. As a specific example, we identify one gene–*TCOF1*, responsible for a syndromic form of congenital hearing loss–in which deleterious variants are also associated with adult-onset hearing loss. We also identify four additional novel candidate genes (*COL5A1*, *HMMR*, *RAPGEF3*, and *NNT*) in which rare variant burden may be associated with hearing loss. Our results confirm that rare variants in Mendelian hearing loss genes contribute to polygenic risk of hearing loss, and emphasize the utility of healthcare system cohorts to study common complex traits and diseases.

## Author summary

Age-related hearing loss is relatively common and has a genetic component to risk, though little is known about specific genes that are involved. Here, we study participants in the Penn Medicine BioBank to identify factors that increase risk of hearing loss. We show that loss-of-function variants in genes that are known to cause Mendelian forms of hearing loss also increase risk of age-related hearing loss. Because many such genes have been identified, they can be used to interpret the results of genome-wide association studies and to investigate the biological basis of age-related hearing loss. Our results also emphasize that many reported Mendelian hearing loss variants are incompletely penetrant and may act cumulatively. Finally, our study shows how hospital-recruited biobank cohorts can aid in the study of common conditions like hearing loss, even when those conditions are not the primary reason for contact with the health system.

## Introduction

Hearing loss (HL) is one of the most common age-related conditions. Around 50% of American adults experience some difficulty hearing and 50% of those aged 70 and over have hearing loss to a level that is disabling [1]. Estimates of the heritability of age-related hearing loss range from 30% to 70% [2] and large genome-wide association studies (GWAS) have identified 89 independent loci that are significantly associated with risk of age-related HL though, as with most GWAS results, the effects are small [3–8]. In parallel, extensive work with family-based and exome sequencing studies of patients with congenital hearing loss has identified 124 genes associated with nonsyndromic HL, and 45 associated with syndromic HL [9], although variants in syndromic HL genes can also cause nonsyndromic HL [10]. These variants are usually assumed to act in a Mendelian fashion and are classified as autosomal dominant (DFNA), autosomal recessive (DFNB) or X-linked (DFNX). Prelingual HL is most commonly associated with homozygous variants in DFNB genes whereas heterozygous variants in DFNA genes tend to cause postlingual HL [11,12]. Although age-related and congenital HL are often studied separately, it is clear that there is substantial overlap between genes associated with the two conditions. Heritability and common variant GWAS associations with age-related HL are enriched near congenital HL genes [7,13], and around 25% of loci identified in GWAS for age-related HL overlap with congenital HL genes [3]. Recently, several studies of age-related HL found high burdens of rare or predicted deleterious variants in known HL genes [14–16], an observation that suggests that deleterious variants, or combinations of deleterious variants, in congenital HL genes might contribute to increased risk of age-related HL.

**Fig 1:**
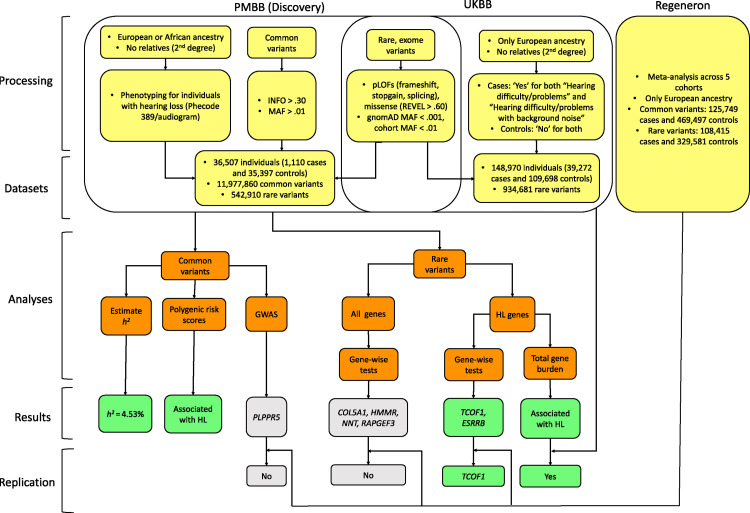
Project workflow, describing data pre-processing such as inclusion/exclusion criteria for individuals and variants, and final datasets used for analyses. Discovery analyses were performed in PMBB for both common and rare variants. Select results were replicated in UK BioBank and using the results of Praveen et al [8].

Unlike children, adult patients are rarely assessed for genetic etiology when presenting with hearing loss. As current clinical management of adult-onset HL would not be significantly impacted by results of genetic testing, such investigation would have low immediate clinical utility. However, understanding the genetic basis of age-related HL may have longer-term benefits for translational therapy, particularly if such therapy can be linked to congenital HL genes, many of which are well-studied in mouse or other models. Greater understanding of HL genetics would also enable screening of high risk individuals at an earlier age who might benefit from preventive or restorative treatment options. Because age-related HL is common, many participants in hospital or healthcare system biobank cohorts are likely to have HL (even if it is not the primary reason for their contact with the health system) providing an opportunity to study the genetic basis of age-related HL in large cohorts without specific recruitment. In this study, we use the Penn Medicine BioBank (PMBB) to investigate the extent to which deleterious variants in known congenital HL genes contribute to polygenic risk of age-related HL and attempt to identify specific genes that contribute to the phenotype (Fig 1).

## Results

### Hearing loss in the Penn Medicine BioBank cohort

PMBB is a database of linked electronic health records (EHR), biospecimens and genetic data of participants recruited through the Penn Medicine health system [17]. The data analyzed here consist of genetic and EHR data from 40,627 individuals. The cohort is 51% male and 49% female, with a median age of 58 years (S1 Table). Based on comparison with genetic reference populations, approximately 75% of the cohort is of European ancestry and 25% is of African American ancestry. A previous analysis of a subset of 16,657 individuals identified an excess of predicted deleterious variants in DFNA genes in participants with audiometrically-defined HL [16].

We first categorized cases and controls following a common scheme in EHR studies. Phenotypes are defined in terms of phecodes–a standardized hierarchical classification of diseases and traits [18,19]. If a patient has two or more instances of a specific phecode in their records, they are a case. If they have zero instances they are a control and if they have one instance they are missing (NA). This approach yielded 3,304 potential cases and 34,704 potential controls. As mild to moderate hearing loss may not always be accurately reflected in EHR data, we cross-checked using a subset of 1,917 individuals for whom we had audiometric data in the form of audiograms, which are measurements of the quietest sound that an individual can hear at a range of frequencies. We find that, of participants with audiograms, 65% of phecode-defined cases and 27% of phecode-defined controls have audiogram-defined hearing loss based on a conventional Pure Tone Average (PTA) threshold of >25 (S2 Table). Due to this relatively high rate of case/control misclassification, we adopted a hybrid strategy to maximize accurate identification of cases and number of controls. We performed all association tests on degree of hearing loss (degree HL), defined as a continuous variable with values from 0–4. We assigned degree HL to individuals with audiograms based on PTA ranges (degree HL 0–4 corresponding to PTA 0–15, 16–25, 26–40, 41–55, and 56+). For individuals without audiograms, we assigned degree HL 0 to phecode-defined controls and removed phecode cases and NA. This assignment is conservative in the sense that the true mean degree HL of phecode controls is greater than 0, but we assumed that it is 0 and would therefore tend to underestimate any associations.

### Deleterious variant burden in known HL genes is associated with risk and degree of HL

**Fig 2:**
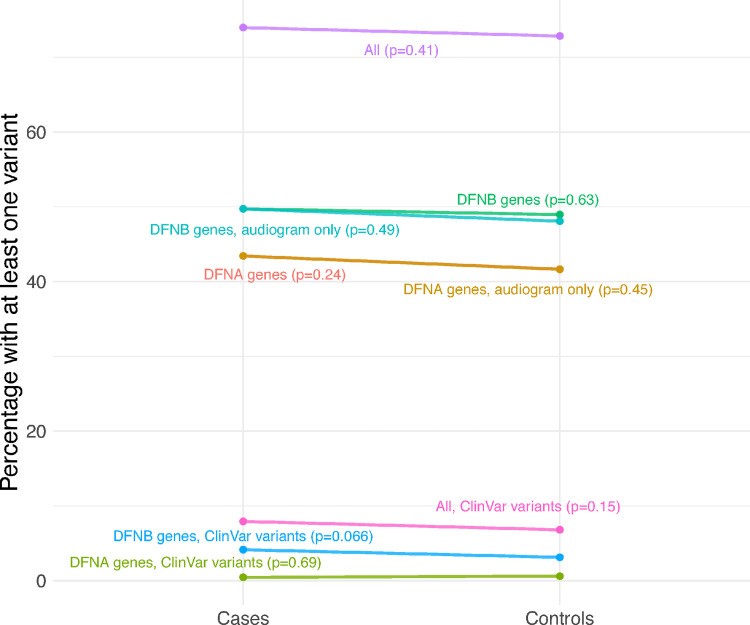
Percentage of hearing loss (HL) cases and controls with at least one variant in different gene and variant categories. “All” refers to the full list of known HL genes, “DFNB” and “DFNA” percentages are only for genes with recessive and dominant forms of Mendelian inheritance, respectively, “audiogram only” percentages are restricted to only individuals with audiograms, and percentages with “ClinVar variants” only included variants with pathogenic/likely pathogenic ClinVar annotations. P-values from Fisher’s exact test of case versus control carrier counts.

**Fig 3:**
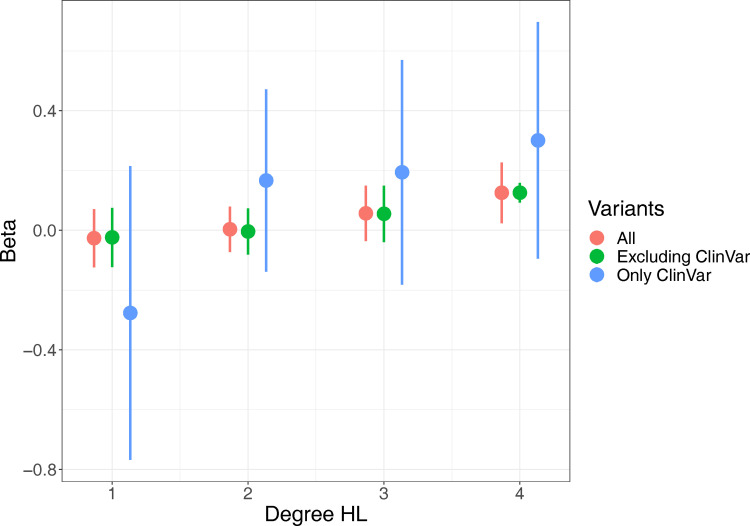
Effect estimates from logistic regression, testing individuals with degree HL 1–4 against individuals with degree HL 0 (including Phecode controls) in known HL genes (bars are 95% CI). Results are split by only including ClinVar variants, excluding ClinVar variants, and using all variants.

Next, we investigated the frequency of predicted deleterious variants in 173 known HL genes (Fig 2, S2 and S3 Tables). We found that 72.8% of 35,397 “controls” (HL 0–1 or Phecode 0) and 74.0% of 1,110 “cases” (HL 2–4) carried at least one deleterious variant in a known HL gene (Fisher’s exact test p = 0.51). Similarly, 6.80% of controls and 7.93% of cases (p = 0.147) carry at least one variant in a known HL gene annotated as “pathogenic” or “likely pathogenic” in ClinVar [20], a database of genetic variants reported to be associated with disease. We regressed degree HL on the burden count, defined as the total number of predicted deleterious variants in known HL genes including sex, age, age^2^ and 20 genomic principal components as covariates, and find that degree HL is associated with burden count (β = 4.8×10^−3^ per-variant, p = 0.03, Table 1). Restricting to subsets of variants we find larger point effects for pathogenic or likely pathogenic ClinVar variants compared to other variants, but the effects of variants in DFNA and DFNB genes were not significantly different. The association is still significant and ten-fold larger in magnitude if we restrict to N = 1,917 individuals with audiograms (β = 4.7×10^−2^ per-variant, p = 0.04). Next, we estimated the effect of burden for each degree HL independently, confirming that gene burden is associated with both the presence and severity of hearing loss (Fig 3). Finally, we replicated this result in UK BioBank (UKB) using binary hearing loss as a phenotype since audiograms were not available (p = 1.84×10^−12^ for association between loss-of-function variant burden in known HL genes and HL). Though absolute effect sizes are not directly comparable between UKB and PMBB because of the different phenotypes, we noted that in UKB, as in PMBB, the effect of each ClinVar variant was four times the size of each non-ClinVar variant, the effects of variants in DFNA and DFNB genes were similar, and the effects of variants in genes that can act as both DFNA and DFNB were larger (Table 2). Taken together, these results demonstrate that increased burden of known and predicted deleterious variants in Mendelian HL genes is associated with increased risk and severity of adult-onset HL.

**Table 1:** Association of total burden in known hearing loss genes in PMBB with degree HL.

	Beta	SE	P
**All variants**	0.00483	0.00224	0.0308
**ClinVar variants**	0.0179	0.00975	0.0660
**Non-ClinVar variants**	0.00467	0.00228	0.0403
**Variants in DFNA genes**	0.00701	0.00344	0.0415
**Variants in DFNB genes**	0.00269	0.00314	0.392
**Variants in DFNA+B genes**	0.00687	0.00820	0.403

**Table 2:** Association of total burden in known hearing loss genes with HL in UKB.

	Beta	SE	P
**All variants**	0.041	0.0059	1.84×10^−12^
**ClinVar variants**	0.141	0.0248	1.32×10^−08^
**Non-ClinVar variants**	0.036	0.0060	4.11×10^−09^
**Variants in DFNA genes**	0.039	0.0094	3.19×10^−05^
**Variants in DFNB genes**	0.034	0.0081	2.40×10^−05^
**Variants in DFNA+B genes**	0.098	0.0204	1.79×10^−06^

### Deleterious variants in *TCOF1* are associated with age related HL

**Fig 4:**
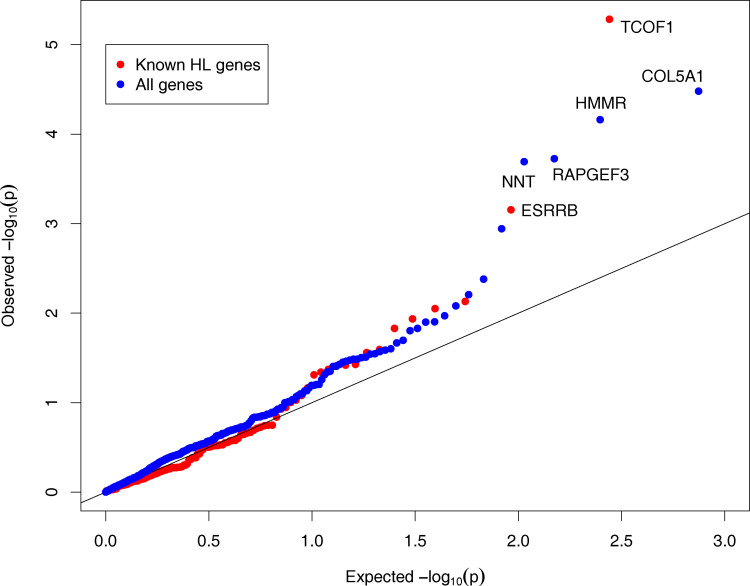
QQ-plot for association of deleterious variant burden with degree HL in 173 known HL genes (red, case carriers > 0) and across 373 genes not previously associated with hearing loss (blue, case carriers > 25). Genes significant at FDR<0.05 are labeled. Genomic inflation factor λ = 0.78 for known HL genes and 1.44 for genes not previously associated.

We tested for association between predicted deleterious variant burden of individual HL genes, and degree HL, using all individuals (S3 Table). We found one significant gene, *TCOF1*, at Benjamini-Hochberg false discovery rate (FDR) < 0.05 (FDR = 7.2×10^−4^, p = 5.2×10^−6^) (Fig 4, Table 3). We replicated this association in a previously reported case-control study of self-reported HL by Praveen et al. which performed gene burden analysis in approximately 108,000 cases and 330,000 controls [8] (minimum p = 8.0×10^−4^ over 24 burden models, S4 Table). The next most significant gene in our data was *ESRRB* (FDR = 0.06, p = 7.0×10^−4^), a nuclear receptor associated with autosomal recessive hearing loss [21]. However, this association did not replicate (minimum p = 0.011 over 24 burden models, S4 Table).

**Table 3:** Known hearing loss gene burden associated with degree hearing loss (FDR < 0.1) in PMBB.

Gene	Beta	SE	P	Carriers	Carrier Freq.	FDR
*TCOF1*	0.798	0.175	5.20×10^−6^	8	0.00022	0.0008788
*ESRRB*	0.148	0.0434	7.00×10^−4^	129	0.0035	0.05915

Autosomal dominant mutations in *TCOF1* cause Treacher Collins Syndrome, which involves craniofacial deformities and conductive hearing loss due to abnormal neural crest cell development [22,23]. The majority of Treacher Collins Syndrome cases are caused by truncating mutations that result in *TCOF1* haploinsufficiency. We identified 8 carriers of predicted loss-of-function or pathogenic missense variants in our dataset (S5 Table). None of these variants were previously reported in ClinVar. Of these carriers, 2 had moderate to severe hearing loss with degrees 3 and 4, but only one (the individual with HL 4) had a clinical diagnosis of Treacher Collins Syndrome. After manually reviewing charts of all carriers, we found that an additional three individuals had reports of hearing loss, and one other individual reported tinnitus. Therefore, 4/7 carriers without a clinical diagnosis of Treacher Collins syndrome had some evidence of hearing impairment. None of these individuals’ charts reported evidence of craniofacial deformities or other Treacher Collins symptoms. These results demonstrate that damaging variants in *TCOF1* that do not manifest in Treacher Collins Syndrome may nonetheless increase risk or severity of hearing loss.

### Four candidate novel HL-associated genes

We next searched for genes contributing to adult-onset HL but are not known to cause congenital HL–i.e., were not on our list of 173 known HL genes. We tested for association of individual gene burden with degree of hearing loss, using the same model as for the total gene burden in previous sections. In initial exome-wide results, we observed extreme inflation in test statistics. Randomizing the phenotype did not reduce this inflation indicating that it was due to poor calibration of test statistics rather than uncorrected population stratification [e.g., 17,24]. We therefore filtered genes using a threshold of >25 case carriers, which restricted analyses to a set of 373 genes, and produced well-calibrated test-statistics.

**Fig 5:**
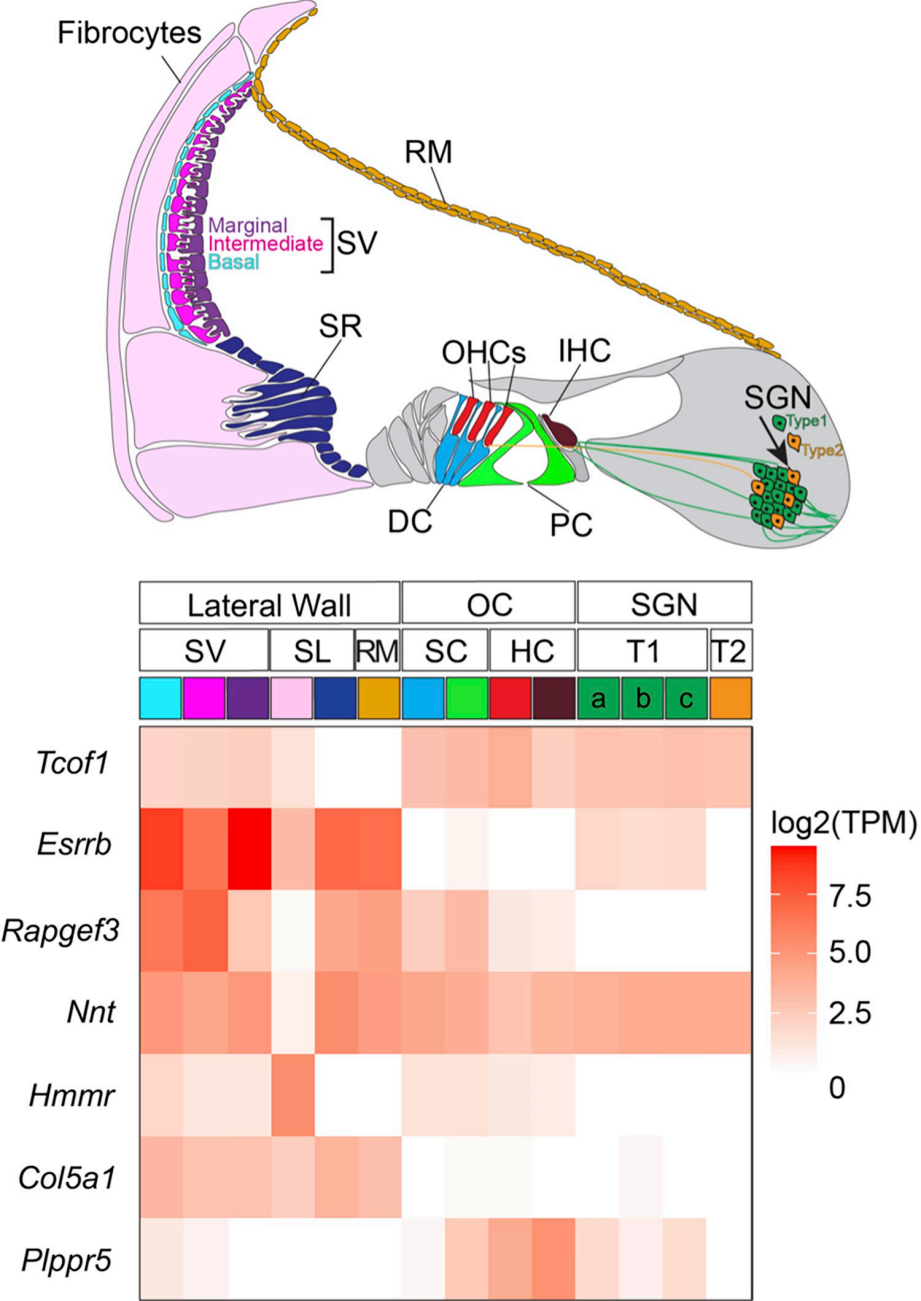
Expression of genes identified in our study in specific cell types from a representative transverse section through the mouse cochlea. The heat map was generated from previously published bulk and single cell RNA-seq datasets deposited in the gEAR portal (https://umgear.org/). Abbreviations: Deiters’ cells (DC), hair cells (HC), inner hair cell (IHC), outer hair cells (OHCs), pillar cells (PC), Reissner’s membrane (RM), spiral ganglion neurons (SGN) Type 1 (T1) and Type 2 (T2), spiral ligament (SL), spindle-root cells (SR), stria vascularis (SV), support cells (SC).

Of these 373 genes, four (*COL5A1*, *HMMR*, *RAPGEF3*, and *NNT*) were significant at FDR < 0.05 (Tables 4 and S6, Fig 4). None of these genes were significantly associated with binary HL in the Praveen et al. study [8] (S4 Table). Nonetheless, all four genes are expressed in cell types in the mouse cochlea that are relevant for hearing (Fig 5). Moreover, several of these genes have additional evidence in humans or mice that supports a role in hearing loss. In particular variants in *COL5A1*, which encodes a subunit of type V collagen, are associated with Ehlers-Danlos syndrome, a connective tissue disorder that may also involve the auditory system, including conductive and sensorineural hearing loss [25–27]. *RAPGEF3*/*EPAC1* is a cAMP sensitive guanine nucleotide exchange factor for the small GTPases RAP1 and RAP2. *Rapgef3/Epac1* knockout mice display pancreatic beta-cell dysfunction and metabolic syndrome [28], which are known risk factors for sensorineural hearing loss [29]. *Rapgef3/Epac1* is also upregulated in response to noise in rats and its pharmacological inhibition has been shown to attenuate inner ear pathology caused by noise exposure [30]. The *nicotinamide nucleotide transydrogenase* (*NNT*) gene encodes an integral protein of the inner mitochondrial membrane. Mice with *Nnt* mutations exhibit impaired insulin secretion, which is also known to increase risk of hearing loss [31,32]. Thus, plausible causal mechanisms may be inferred for 3 out of 4 candidate novel genes associated with adult-onset HL that were identified in our rare variant gene burden analysis.

**Table 4:** Novel gene burden associated with degree hearing loss (FDR < 0.05) in PMBB.

Gene	Beta	SE	p	Carriers	Carrier Freq.	FDR
*COL5A1*	0.0586	0.0141	3.31×10^−5^	1255	0.034	0.0123
*HMMR*	0.0974	0.0245	6.89×10^−5^	407	0.011	0.0128
*RAPGEF3*	0.0731	0.0196	1.88×10^−4^	647	0.018	0.0189
*NNT*	0.0507	0.0136	2.03×10^−4^	1013	0.028	0.0189

### Genome-wide association analysis identifies *PLPPR5* as a single candidate locus

**Fig 6:**
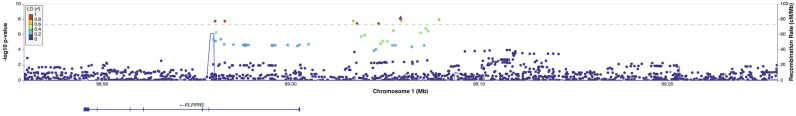
Local association plot of GWAS results at the *PLPPR5* locus (generated with LocusZoom [48]).

We conducted common variant GWAS under an additive model in all study individuals, for variants with a minor allele frequency greater than 1%. As with the burden analysis, we randomized the phenotype and observed quantile-quantile plots (QQ-plots) of the randomized and observed results, and increased the allele frequency cutoff until inflation was not observed in the randomized results (genomic inflation constant λ = 1.01). We identified a single genome-wide significant locus upstream of *PLPPR5* (lead SNP chr1:99058420:C:T, MAF = 0.012, p = 8.27×10^−9^, Fig 6). *PLPPR5* has previously not been associated with hearing loss–nevertheless, single cell RNA-seq analysis of mouse cochlea does indicate selective *Plppr5* expression in inner hair cells (Fig 5). Mouse knockout of *Plppr5* does not result in HL [33], although hearing was only measured in young mice, so an age-related HL phenotype would not be revealed in this analysis. This association did not replicate in the Praveen et al. GWAS results (p = 0.759) [8] and would need to be replicated elsewhere before being considered reliable. Conversely, of the 53 genome-wide significant lead variants reported in that study, 45 were present in our dataset of which 9 had p-values <0.05, significantly more than expected by chance (binomial p-value 0.0003, S7 Table). We also replicated at p<0.05 2 out of 12 novel rare variants identified by Ivarsdottir et al. [5] that were also present in our dataset (S8 Table), which is not significantly more than expected by chance (p = 0.11) but unsurprising since we have low power for rare variant associations.

### Polygenic score and rare variant burden score have low predictive power

Finally, we computed polygenic risk scores (PRS) using PRS-CS [34] and a previously reported GWAS of hearing loss in UK BioBank [35]. The PRS is constructed by summing up all the effects of all the GWAS-identified variants carried by an individual and provides an estimate of their genetic risk. We summarize the predictive power of this score using the incremental *R*^*2*^ –the difference in *R*^*2*^ between a linear model of degree HL with and without the PRS (but including other covariates, Methods). The incremental *R*^*2*^ of the PRS is 0.19% (bootstrap standard error 0.054%), rising to 1% if we restrict to individuals with audiogram data (Table 5). As observed for other traits [36], the predictive power of the PRS is much lower in individuals of African American ancestry, compared to those of European ancestry. The predictive power of the rare variant burden score is much lower than the PRS (Table 5), and also lower in African American compared to European ancestry individuals. This is surprising because, unlike the PRS, the burden score was not directly trained in a European ancestry cohort. Despite these low incremental *R*^*2*^, both scores have the potential to identify a subset of individuals at high risk of HL. For example, individuals in both the top 10% of the PRS distribution and the top 10% of HL gene burden distribution had an odds ratio of 2.9 (for degree HL 2 or greater), similar to individuals in the top 1% of the PRS distribution (odds ratio 2.6).

**Table 5:** Predictive power (incremental *R*^*2*^) of polygenic risk scores and burden of deleterious variants in known HL genes for degree HL in PMBB.

	All individuals			Audiogram only		
Ancestry	All	EUR	AFR	All	EUR	AFR
**PRS**	0.153%	0.186%	0.032%	0.970%	1.00%	0.236%
**HL burden**	0.012%	0.017%	0.003%	0.166%	0.274%	0.000%

## Discussion

Our study illustrates both the opportunities and challenges of using EHR data to study a common complex condition like hearing loss. In particular, the subset of individuals in our data with audiograms makes it clear that EHR-derived phenotypes are highly inaccurate for this phenotype, perhaps because phecodes are largely constructed using hospital billing codes and hearing loss is not typically the primary reason for hospital visits. Published large GWAS studies have relied largely on self-reported hearing loss in UK Biobank [3,5] which may be similarly inaccurate, though some studies include a subset of individuals with audiograms. From an analysis perspective, it may make more sense to think of audiogram measurements, self-reported HL and EHR-derived HL as different, though related, phenotypes. For example, our stricter “case” definition entails a case proportion of 3.0% in PMBB, compared to 26.4% in UK Biobank. Because we had audiometric data only for a relatively small proportion of our cohort, we actually used a hybrid strategy where audiogram data identified “cases” and people with neither audiograms nor EHR-derived HL were assumed not to have hearing loss (i.e., “controls”, HL 0). We chose this strategy because it maximizes sample size while being conservative, in the sense that it would tend to underestimate associations with HL. Individuals without EHR-derived HL but with audiograms have a mean HL of 0.8 on our 0–4 scale, providing one estimate of the true baseline level in these individuals and suggesting that our effect sizes estimates would be biased downwards. On the other hand, this could be an overestimate relative to the general population since it is possible that people who have audiograms are enriched for people with some hearing loss. Ultimately, the limited number of individuals with audiometric data is probably the major factor limiting the power of our study. One strategy that other studies have used is to analyze audiogram and self-reported data separately and to then meta-analyze the results [5]. It remains unclear what the best way to combine these phenotypes is. This heterogeneity in phenotype may contribute to the lack of replication of some of our findings in other datasets and collecting larger datasets of genotyped individuals with audiometric data should be a priority for further research.

We identified and replicated a novel association of *TCOF1* variants with adult-onset HL. While *TCOF1* is known to cause congenital HL as part of Treacher-Collins syndrome, it has not previously been reported to increase risk of adult-onset HL. While hearing loss in Treacher-Collins syndrome is generally conductive due to malformation of middle ear structures, in at least one of our *TCOF1* cases hearing loss is sensorineural, suggesting a different mechanism. This result is an example of how knowledge about genes associated with early-onset or developmental conditions can be used to prioritize gene discovery for related adult-onset conditions. Similar observations have been made about the excess contribution of genes that are essential or involved in developmental disorders to psychiatric traits such as autism [37] and schizophrenia [38].

Our observation that rare variant burden in known HL genes is associated with both risk and severity of adult onset HL supports previous observations that common variant heritability is enriched around known HL genes [13] and that deleterious variants in congenital HL genes are associated with age-related HL [14–16]. Conversely, the high rate of predicted deleterious coding mutations in controls emphasizes that many of these variants have low penetrance [14], and their effects on age-related HL are cumulative and polygenic, rather than Mendelian. Further, the low predictive power of both rare variant burden and polygenic scores suggests that these are not yet particularly useful tools for HL risk prediction. One limitation of our analysis is that we cannot distinguish the extent to which the polygenic contribution to disease from congenital HL genes is uniformly distributed across those genes, as opposed to being concentrated in a relatively small number of genes which we lack power to identify individually.

We also identified five novel genes potentially associated with HL–four through gene burden analysis and one through GWAS. Although these genes are all biologically plausible, these associations are only modestly supported and none of them replicated in the Praveen et al. study. We therefore consider these associations as only candidates until replicated. One limitation is that Praveen et al. used self-reported hearing loss as opposed to audiometric data. Another is that for rare variants we only looked at coding variation and not regulatory variation at these genes, which might explain a larger proportion of heritability.

Another opportunity and challenge of our dataset is the level of ancestral diversity. Approximately 25% of the individuals in the Penn Medicine BioBank are of African American ancestry. Performing genetic discovery within and across cohorts of diverse ancestry is both a key goal and largely open question in human genetic research today [39,40]. While previous studies have meta-analyzed ancestry-specific results [17], we instead analyzed all individuals together, correcting for ancestry using genome-wide principal components. Exome-based studies may in fact be more amenable to cross-ancestry analysis since they assume that the loss-of-function variants that are counted in the test are in fact the causal variants. Therefore, unlike GWAS these tests should not be affected by differences in linkage disequilibrium and tagging of causal variants across populations. We expected that the effect of coding variants would be similar across ancestries. However, the observation that the predictive power of rare variant burden on HL is lower in African American ancestry individuals compared to European ancestry individuals (like polygenic scores [36]) calls this expectation into question and requires further investigation.

In summary, we used biobank data and a prioritized list of known HL genes to identify novel associations with adult-onset HL. We showed that burden of deleterious variants in known HL genes is robustly associated with HL and identified five additional candidate genes through exome- and genome-wide analysis. Because hearing loss is so common in older individuals, healthcare cohorts like the Penn Medicine Biobank have great potential to allow gene discovery, and validation of predictive tests, even if not specifically collected for that purpose, and we expect that these approaches will become increasingly common as such cohorts expand in size.

## Methods

### Ethics statement

The collection, storage and analysis of biospecimens, genetic data and data derived from electronic health records as part of the Penn Medicine BioBank is approved under University of Pennsylvania IRB protocol #813913. Each participant provided written informed consent.

### Study setting and participants

The Penn Medicine BioBank (PMBB) has recruited approximately 60,000 participants and hosts data from patients of clinical practice sites of the University of Pennsylvania Health System. Each participant provided informed consent regarding storage of biological specimens, genetic sequencing, and access to all available EHR data. This study was approved by the Institutional Review Board of the University of Pennsylvania.

### Data collection

Exome sequences were generated by the Regeneron Genetics Center (Tarrytown, NY). Sequencing was performed in 2020 with the custom IDT xGen v1 exome capture platform and sequenced on an Illumina NovaSeq 6000 system using S4 flow cells. Sequences were mapped and variants were annotated using ANNOVAR, gnomAD, and REVEL (Rare Exome Variant Ensemble Learner), and samples with low exome sequencing coverage (less than 85% of targeted sites at 20X coverage), apparent contamination (D-statistic > 0.4), discordance with reported sex, or that were duplicates, were removed, as previously described [17,41]. After variant calling using the weCall variant caller, we set any site with fewer than seven reads to missing and required variants to have at least one homozygote call or at least one heterozygote call supported by at least 15% of reads. We inferred ancestry by projecting array genotype data onto principal component axes defined by individuals from the 1000 Genomes Project [42] and fitting a Gaussian mixture model. We also removed individuals such that all were unrelated up to the 3^rd^ degree. A total of 9,356 individuals considered African American ancestry and 27,151 individuals considered European ancestry were included after quality control measures including removal of individuals with missing covariates.

### Phenotyping

We extracted audiometric data from a clinical database (AudBase) of audiograms performed at the Hospital of the University of Pennsylvania audiology practice between May 5, 2013 and February 25, 2021–1,917 audiograms were available for the 36,507 study individuals. From the clinical database, we calculated pure-tone average (PTA) for bone and air conduction using the arithmetic mean of the hearing threshold in decibels at 500, 1000, and 2000 Hertz. We recorded air conduction PTA of the worse ear and assigned HL levels according to previously published categorization: PTA 0–15 (degree HL 0), PTA 16–25 (degree HL 1, mild), PTA 26–40 (degree HL 2, moderate), PTA 41–55 (degree HL 3, severe), and PTA 56+ (degree HL 4, profound) [43]. To identify additional controls phecode 389 (Hearing Loss) was used, counting individuals as controls if they had zero EHR records of this phecode. In total, we included 35,397 controls and 1,110 hearing loss cases in analyses (with degree HL for cases broken down as N = 496 degree HL 0, N = 311 degree HL 1, N = 516 degree HL 2, N = 334 degree HL 3, and N = 260 degree HL 4).

### Rare variant gene burden

To select variants to include in gene burden analysis, we used a combination of minor allele frequency filters and predicted pathogenicity. First, we removed variants if their gnomAD allele frequency in African and Non-Finnish European individuals was greater than .001. We then filtered remaining variants on an allele frequency of .01 in the entire dataset. Next, we restricted to predicted loss-of-function (pLoF) variants, namely frameshift mutations, stop-gains, and those that disrupt canonical splice sites. We also included nonsynonymous missense variants after filtering to restrict to those with high predicted pathogenicity, defined as the Rare Exome Variant Ensemble Learner (REVEL) score being greater than 0.6 [41]. We annotated variants based on their ClinVar annotation, considering known pathogenic variants to be those with annotation “Pathogenic”, “Likely pathogenic”, or “Pathogenic/Likely pathogenic”. Ultimately, we identified 11,641 predicted deleterious variants, 575 of which were reported pathogenic variants. For known HL genes, we included 173 autosomal genes, of which 66 were DFNA, 97 DFNB, and 10 had multiple inheritance patterns. For the gene burden analyses, any included variants in a gene were summed to create a single gene burden for that individual using BioBin [44], which we used in downstream association analyses. For the total gene burden analyses in known HL genes, we summed together all such variants in known HL genes for each individual. We tested for association using linear regression with degree HL as the outcome and including age, age^2^, sex, and genome-wide PCs 1–20 as covariates.

We performed permutation analyses to determine the (case) carrier cut-off and minor allele frequency thresholds for the burden tests, as we observed test statistic inflation in the QQ-plots of the -log_10_(p) from the association tests without additional filtering. Briefly, we randomly permuted the phenotype and re-ran the association tests. For known HL genes, we assumed that variants in these genes would be deleterious, as deleterious variants in these genes were how they were discovered and thus we filtered based on case carriers >0 instead of thresholding based on carriers only. When expanding analyses to all genes, we again used a case carrier threshold, but were much more conservative as we were attempting novel discovery–we increased the case carrier threshold in increments of 5 until we observed very minimal test statistic inflation in the randomly permuted -log_10_(p) QQ-plot, and chose a threshold of >25 case carriers. To correct for multiple comparisons, we treated burden in known and novel HL genes separately, and used Benjamini-Hochberg correction.

### Common variants

Array genotypes were processed according to these specifications (https://pmbb.med.upenn.edu/data-access/data-release/2020_2_0/imputed/), and were imputed to the TOPMed reference panel [45] using the Michigan Imputation Server [46]. Common variant analysis was performed on the same set of individuals that were used for the rare variant burden analysis. We filtered variants on imputation *R*^*2*^ >.30 and used the same modeling setup and covariates as with the burden tests. We performed association tests using Plink 1.9 [47]. To determine the minor allele frequency threshold, we ran similar permutation analyses for GWAS of common variants as we did with the burden analyses. After randomly permuting the phenotype, we started at a minor allele frequency threshold of .1%, increased it to .5%, and observed minimal test statistic inflation at a threshold of 1%.

### Polygenic risk scores

We calculated PRS for PMBB individuals with PRS-CS [34] using default settings, including restricting SNPs to a set of 1,009,605 HapMap3 SNPs. We used European ancestry GWAS summary statistics from UKB [35]. We report incremental *R*^*2*^, calculated by subtracting the difference in *R*^*2*^ between HL regressed on all covariates (age, age^2^, sex, PCs 1–20) and PRS from the *R*^*2*^ when regressing HL just on the covariates.

### Replication

We replicated the association between gene burden and HL in UK Biobank. Hearing loss cases were defined as those who answered ‘Yes’ for both “Hearing difficulty/problems” and “Hearing difficulty/problems with background noise”, and controls were individuals who answered ‘No’ for both–remaining individuals were removed. We note that this phenotyping definition is similar to previous analyses of hearing loss in UKB [3]. 148,970 (39,272 cases and 109,698 controls (26.4% cases)) European ancestry participants with available exome sequencing were included. We defined predicted deleterious variants as above and performed logistic regression including age, age^2^, sex, and the top 5 genetic principal components provided by UKB.

We replicated novel significant gene and GWAS associations using results from Praveen et al. [8]. Briefly, this study reports a meta-analysis of five cohorts of European ancestry, including UK Biobank, with 125,749 cases and 469,497 for common variants and 108,415 cases and 329,581 controls for rare variant burden analyses. Since the phenotype and variant definitions were different between this study and our dataset, we tested 24 different models per gene (6 different allele frequency thresholds and 4 definitions of variant deleteriousness), and considered a gene to replicate if the minimum P-value was below a Bonferroni-corrected significance threshold of 0.05/24.

## Supporting information

S1 Table Basic demographic properties of PMBB cohort. (XLSX)

S2 Table Comparison between Phecode-defined case/control status and Audiograms. (XLSX)

S3 Table List of 173 known congenital hearing loss (HL) genes and their Mendelian inheritance pattern(s). (XLSX)

S4 Table Replication results for known and novel HL genes identified through LoF burden association. (XLSX)

S5 Table LoF and predicted deleterious TCOF1 mutations. (XLSX)

S6 Table LoF burden association results for 373 genes not previously reported as HL genes with at least 25 carriers. (XLSX)

S7 Table Attempted replication results for associations reported by Praveen et al. [8].
(XLSX)

S8 Table Attempted replication results for novel associations reported by Ivarsdottier et al. [5].
(XLSX)

S1 Note Members of the Penn Medicine BioBank Consortium. (PDF)
