# Supplementary material for: Gene burden analysis identifies genes associated with increased risk and severity of adult-onset hearing loss in a diverse hospital-based cohort
Source: PLoS Genet. 2023 Jan 19;19(1):e1010584. doi: 10.1371/journal.pgen.1010584 (PMC9888707; doi:10.1371/journal.pgen.1010584)
Supplement: S1 Note — (PDF) [file pgen.1010584.s009.pdf]

Penn Medicine BioBank Banner Author List and Contribution Statements

PMBB Leadership Team
Daniel J. Rader, M.D., Marylyn D. Ritchie, Ph.D., Michael D. Feldman M.D.

Contribution: All authors contributed to securing funding, study design and oversight. All authors reviewed the final version of the manuscript.

Patient Recruitment and Regulatory Oversight
JoEllen Weaver, Nawar Naseer, Ph.D., M.P.H., Afiya Poindexter, Ashlei Brock, Khadijah Hu-Sain, Yi-An Ko

Contributions: JW manages patient recruitment and regulatory oversight of study. NN manages participant engagement, assists with regulatory oversight, and researcher access. AP, AB, KH, YK perform recruitment and enrollment of study participants.

Lab Operations
JoEllen Weaver, Meghan Livingstone, Fred Vadivieso, Ashley Kloter, Stephanie DerOhannessian, Teo Tran, Linda Morrel, Ned Haubein, Joseph Dunn

Contribution: JW, ML, FV, SD conduct oversight of lab operations. ML, FV, AK, SD, TT, LM perform sample processing. NH, JD are responsible for sample tracking and the laboratory information management system.

Clinical Informatics
Anurag Verma, Ph.D., Colleen Morse, M.S., Marjorie Risman, M.S., Renae Judy, B.S.

Contribution: All authors contributed to the development and validation of clinical phenotypes used to identify study subjects and (when applicable) controls.

Genome Informatics
Anurag Verma Ph.D., Shefali S. Verma, Ph.D., Yuki Bradford, M.S., Scott Dudek, M.S., Theodore Drivas, M.D., PH.D.

Contribution: A.V., S.S.V. are responsible for the analysis, design, and infrastructure needed to quality control genotype and exome data. Y.B. performs the analysis. T.D. and A.V. provides variant and gene annotations and their functional interpretation of variants.
